# Effect of COVID-19-Related Interventions on the Incidence of Infectious Eye Diseases: Analysis of Nationwide Infectious Disease Incidence Monitoring Data

**DOI:** 10.3389/ijph.2022.1605211

**Published:** 2022-10-20

**Authors:** Woo-Ri Lee, Li-Hyun Kim, Gyeong-Min Lee, Jooyoung Cheon, Young Dae Kwon, Jin-Won Noh, Ki-Bong Yoo

**Affiliations:** ^1^ Division of Cancer Control and Policy, National Cancer Control Institute, National Cancer Center, Goyang, South Korea; ^2^ Department of Healthcare Institution Support, National Health Insurance Service, Wonju, South Korea; ^3^ Department of Family Medicine, College of Medicine, Chung-Ang University, Seoul, South Korea; ^4^ Department of Nursing Science, Sungshin Women’s University, Seoul, South Korea; ^5^ Department of Humanities and Social Medicine, College of Medicine and Catholic Institute for Healthcare Management, The Catholic University of Korea, Seoul, South Korea; ^6^ Division of Health Administration, College of Software and Digital Healthcare Convergence, Yonsei University, Wonju, South Korea

**Keywords:** COVID-19, social distancing, interrupted time series, infectious eye disease, segmented regression, nationwide infectious disease incidence monitoring data

## Abstract

**Objective:** Social distancing has been confirmed to reduce the incidence of not only the COVID-19, but also the incidence of other diseases. Therefore, this study aimed to investigate the effect of social distancing policies on the incidence of infectious eye diseases by monitoring their nationwide incidence data in all age groups.

**Methods:** In this study, to analyse the impact of COVID-19 policy on IEDSC, the time periods were divided into two interventions. The first intervention was the first COVID-19 patient report in Korea on 19 January 2020. The second intervention was relaxation of the social distancing policy on 6 May 2020. Segmented regression analysis of the interrupted time series was used to assess COVID-19 policies on the IEDSC.

**Results:** After the first incidence of a COVID-19 patient, IEDSCs decreased significantly in all age groups, while the relaxation of the social distancing policy increased IEDSCs significantly, mostly in all groups.

**Conclusion:** In the post-COVID-19 era, we hope that national-level interventions such as reducing air pollution and employing precautionary measures will significantly reduce the financial burden of developing infectious ophthalmic diseases.

## Introduction

In December 2019, pneumonia caused by a novel coronavirus was reported in Wuhan, China [1]. After the first case occurred, coronavirus disease 2019 (COVID-19) spread rapidly [2], and on 21 January 2020, the first confirmed case occurred in South Korea [3]. In the early stages of the pandemic, non-pharmaceutical interventions such as mask-wearing, hand hygiene, and social distancing were recommended because of the absence of vaccines and antiviral agents to control COVID-19 [4, 5]. In February 2020, after a super-spreader was validated (31st patient), the number of Korean COVID-19 cases was confirmed, and fatalities increased rapidly [6]. Therefore, the government implemented social distancing by the end of February 2020 to prevent the spread of COVID-19 [4]. Social distancing is a measure to mitigate the epidemic of infectious diseases so as to not exceed the capacity of medical institutions [7]. According to the WHO, “social distancing” is an intervention that maintains a physical distance between people and reduces the number of times people come in close contact to prevent the spread of contagious disease [8]. The measures undertaken include school closures and community isolation [8]. Furthermore, 70% of countries worldwide have implemented these social distancing measures [9]. In South Korea, along with personal-level public hygiene management such as ventilation, sterilization, mask-wearing, and personal hygiene management, social distancing was established by working from home and implementing closures of religious facilities, pubs, and schools [5]. After social distancing, public movement gradually declined [10] and the number of confirmed COVID-19 infections gradually decreased. As a result, the Korean government implemented a relaxed social distancing policy on 6 May 2020 [7]. Relaxed social distancing provides personal-level public health management according to detailed guidelines [11]. Social distancing reduces the incidence of COVID-19 and other diseases [5, 12–16]. Prior studies revealed that the social distancing policy has contributed to reducing paediatric infection by eliminating children’s interactions. These studies demonstrated the important association between non-pharmaceutical interventions and infectious disease transmission [5, 12–14]. Additionally, it has been confirmed that social distancing policies effectively slow the spread of viral respiratory diseases in children [15], and that Enterovirus and all-cause pneumonia decreased during COVID-19 [16].

Viral conjunctivitis is the most common type of infectious conjunctivitis and is caused by adenoviruses, enteroviruses, etc. There are no effective antiviral agents for treating viral conjunctivitis, and treatment for the disease has a supportive role in alleviating the symptoms [17–19]. However, research on infectious eye diseases is important because conjunctivitis may have negative effects on many people, resulting in economic and social burdens. In the United States, six million people are affected annually by conjunctivitis. Over $ 2 billion is spent annually on emergency room visits due to eye diseases, and approximately 28% of emergency room visits are due to conjunctivitis [17, 20]. The burden of illness due to infectious eye disease has gradually increased. Social distancing has reduced the number of patients with other infectious diseases as well as with infectious eye diseases. Therefore, this study aimed to investigate the effect of social distancing policies on the incidence of infectious eye diseases (IED) by monitoring their nationwide incidence data in all age groups.

## Methods

### Data

Infectious Eye Disease Suspected Case (IEDSC) data were obtained from the Infectious Disease Portal of the Korea Centers for Disease Control and Prevention (KCDC) [21]. The KCDC reports several infectious diseases on its website on a weekly basis. Reported infectious eye diseases include epidemic keratoconjunctivitis and acute haemorrhagic conjunctivitis. Suspected cases were defined as the number of patients with epidemic keratoconjunctivitis and acute haemorrhagic conjunctivitis in 80 sample-monitoring hospitals [21, 22]. These data were categorized by age (0–6 years, 7–19 years, 20 years or older) [21]. The study period started from the 1st week (1st week of January) in 2017 to the 35th week (4th week of August) in 2020.

In this study, to analyse the impact of COVID-19 policy on IEDSC, the time periods were divided into two interventions. The first intervention was a COVID-19 patient report in Korea on 19 January 2020. The second intervention was the relaxation of the social distancing policy on 6 May 2020. The Korean social distancing policy is an infectious disease management strategy that minimizes contact between individuals and groups to reduce the spread of infectious diseases [23]. It began on 22 March 2020 and was relaxed on 6 May 2020 [24].

### Variables

The dependent variable in this study was the overall IEDSC (epidemic keratoconjunctivitis and acute haemorrhagic conjunctivitis) and IEDSC data categorized by age (0–6 years old, 7–19 years old, 20 years or older). Log transformation was performed. Month dummy variables were included as covariates to capture the monthly variations.

### Statistical Analysis

Segmented regression analysis of the interrupted time series was used to assess COVID-19 policies on the IEDSC. Segmented regression is a quasi-experimental analysis used to evaluate policy interventions [25]. The regression model is as follows:

${\log(Y}_{t})$ = $\beta_{0}+\beta_{1}$ ⅹ ${time}_{t}$ + $\beta_{2}$ ⅹ $time\;after\;{intervention1}_{t}$ + $\beta_{3\;}ⅹ\;{intervention2}_{t}$ + $\beta_{4\;}ⅹ\;time\;after\;{intervention2}_{t}$ + $\overset{11}{\underset{i=1}{\sum }}\beta_{mi}ⅹ\;month{\;}_{i}$ $+e_{t}$

• Y_t_: IEDSC per 1,000 outpatient visits
• time_t_: basic trend (continuous, unit: week)
• time after intervention 1_t_: the period from week 3 of January 2020; first patient incidence of COVID-19 (continuous, unit: week)
• Intervention 2_t_: week 1 of May 2020; the relaxing of social distancing policy (0, 1)
• time after intervention 1-2_t_: the period after intervention 2 (continuous, unit: week)
• month 1–month 11: indicators for monthly seasonality (0, 1)
• $e_{t}$ : error

The time variables present a basic trend and provide a continuous weekly value (1–191 weeks). The time variable was the baseline trend variable. The time after intervention 1 was 0 before the first incidence of COVID-19 and increased from 1 after. The time after intervention 2 was 0 before week 1 of May 2020 and increased from 1 after the social distancing policy was relaxed.

Since there are many intervention variables related to time, interpretation of the segmented regression analysis is difficult. Therefore, we calculated the relative reduction and marginal effects of the dependent variables. The relative reduction was estimated by comparing the IEDSC of 2020 with the average IEDSC of 2017–2019. The marginal effects of COVID-19 incidence in the 4th week of August 2020, compared to the 3rd week of January 2020, were calculated using the formula $\beta_{2\;}\times 32$ ; wherein, 32 indicates the time difference between the 3rd week of January 2020 (159th week of the total study period, just before the first COVID-19 incidence) and the 4th week of August 2020 (191st week of the total study period). The marginal effects of relaxing the social distancing policy in the 4th week of August 2020, compared to that in the 5th week of April 2020, were estimated using the formula: $\beta_{3}$ + $\beta_{4\;}\times 17$ ; wherein, 17 is the time difference between the 5th week of April 2020 (174th week of the total study period, just before relaxing the social distancing policy) and the 4th week of August 2020 (191st week of the total study period). The marginal effects of both policies in the 4th week of August 2020, compared to those in the 3rd week of January 2020, were calculated using the formula ${\beta_{2\;}\times 32+\beta }_{3}$ + $\beta_{4\;}\times 17$ . For statistical analysis, the PROC AUTOREG in SAS version 9.4 (SAS Institute, Cary, NC, United States) was used. The Newey–West standard error correction was used for heteroscedasticity and autocorrelation correction. The studies involving human participants were reviewed and approved by the Institutional Review Board (IRB) (1041849-202206-SB-112-01).

## Results

**FIGURE 1:**
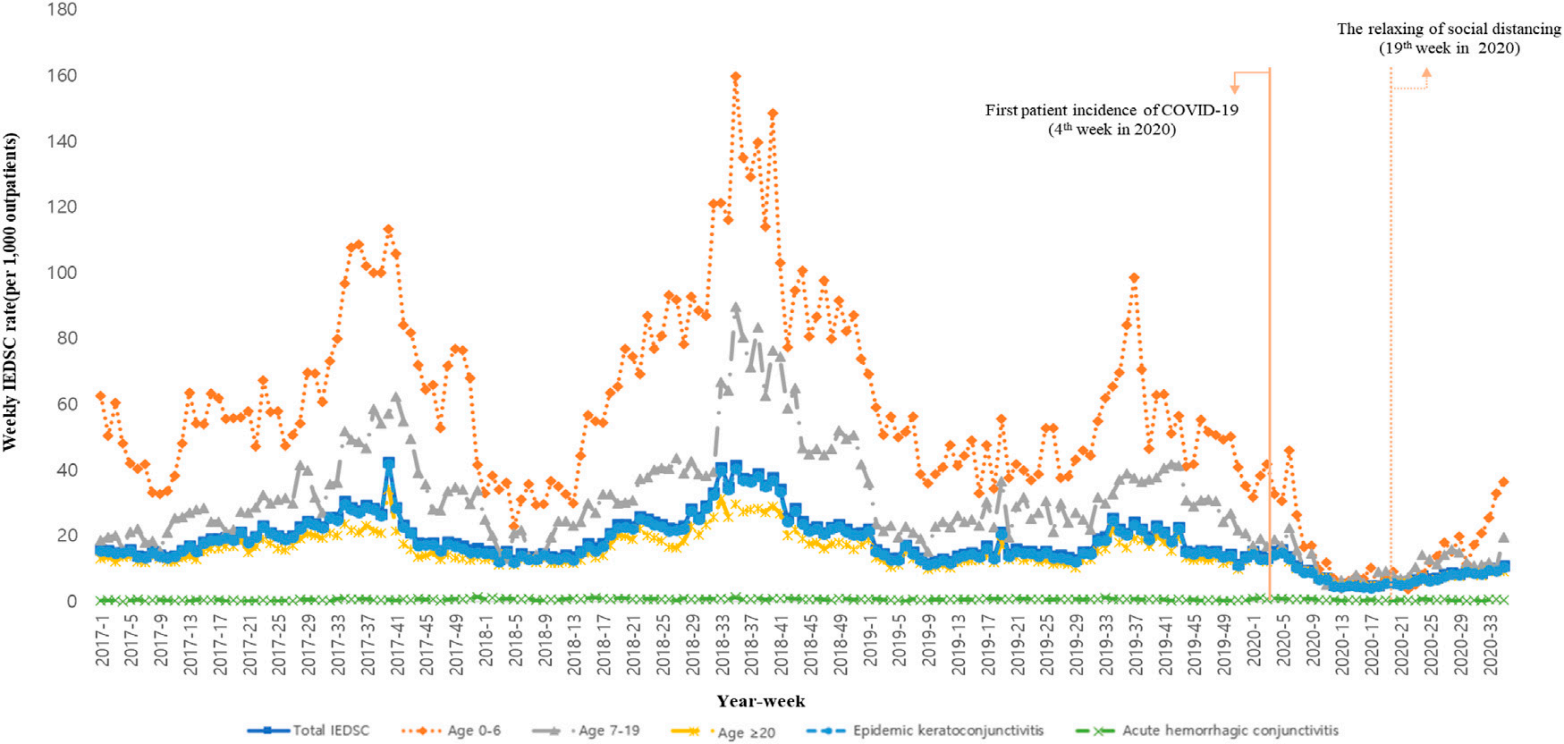
Total incidence of infectious eye disease suspected case and subgroup time trends (South Korea, 2017–2020).

Figure 1 shows the time trends of outpatients per 1,000 for each age group of the IEDSC. According to the overall trend, the number of patients increased rapidly in summer, exhibiting seasonal characteristics. We observed that this trend decreased in spring and winter. The first incidence of COVID-19 in a patient was on 19 January 2020, showed that the weekly IEDSC rate was below 20. In addition, the relaxation of the social distancing policy showed that the number of weekly IEDSC had gradually increased.

An interrupted time-series analysis was performed to check the effect of the two events related to COVID-19 (Table 1). Monthly seasonal effects were significant in all age groups and IEDSC diseases. After the first COVID-19 patient incidence, IEDSCs decreased significantly in all age groups and in all diseases. Relaxing the social distancing policy significantly increased IEDSCs, mostly in all groups.

**TABLE 1:** Interrupted time series analysis to identify the effect of coronavirus disease 2019 on the incidence of infectious eye disease suspected case (South Korea, 2017–2020).

Variable	Total IED	0–6 age	7–19 age	≥20 age	Epidemic keratoconjunctivitis	Acute haemorrhagic conjunctivitis
				Coefficient		
Time	−0.002***	−0.002***	−0.001*	−0.001***	−0.002***	0.003***
First patient incidence of COVID-19						
Time after event	−0.072**	−0.135**	−0.099**	−0.063**	−0.074**	−0.056**
Relaxing of social distancing policy						
Policy	0.422**	0.664*	0.885***	0.358**	0.440**	0.411
Time after policy	0.004***	0.011***	0.005***	0.004***	0.004***	0.002*
Month 1 (Ref)						
Month 2	−0.046	−0.104	−0.047	−0.028	−0.042	−0.087
Month 3	−0.134**	−0.206	0.058	−0.144**	−0.132**	−0.125
Month 4	0.039	0.123	0.276***	−0.010	0.040	0.110
Month 5	0.183*	0.127	0.256**	0.159*	0.192*	−0.102
Month 6	0.268***	0.331*	0.506***	0.197*	0.280***	−0.036
Month 7	0.324***	0.409**	0.584***	0.253**	0.339***	0.014
Month 8	0.583***	0.667***	0.708***	0.488***	0.599***	0.158
Month 9	0.671***	0.843***	1.002***	0.551***	0.694***	0.076
Month 10	0.442***	0.560***	0.896***	0.349***	0.460***	−0.004
Month 11	0.204*	0.430***	0.580***	0.120	0.221**	−0.179
Month 12	0.144	0.387***	0.537***	0.063	0.151	−0.019
R-squared	0.86	0.86	0.83	0.84	0.86	0.20

*p* < 0.05 = *, *p* < 0.01 = **, *p* < 0.001 = ***; COVID-19, coronavirus disease-2019; Ref, reference.

**FIGURE 2:**
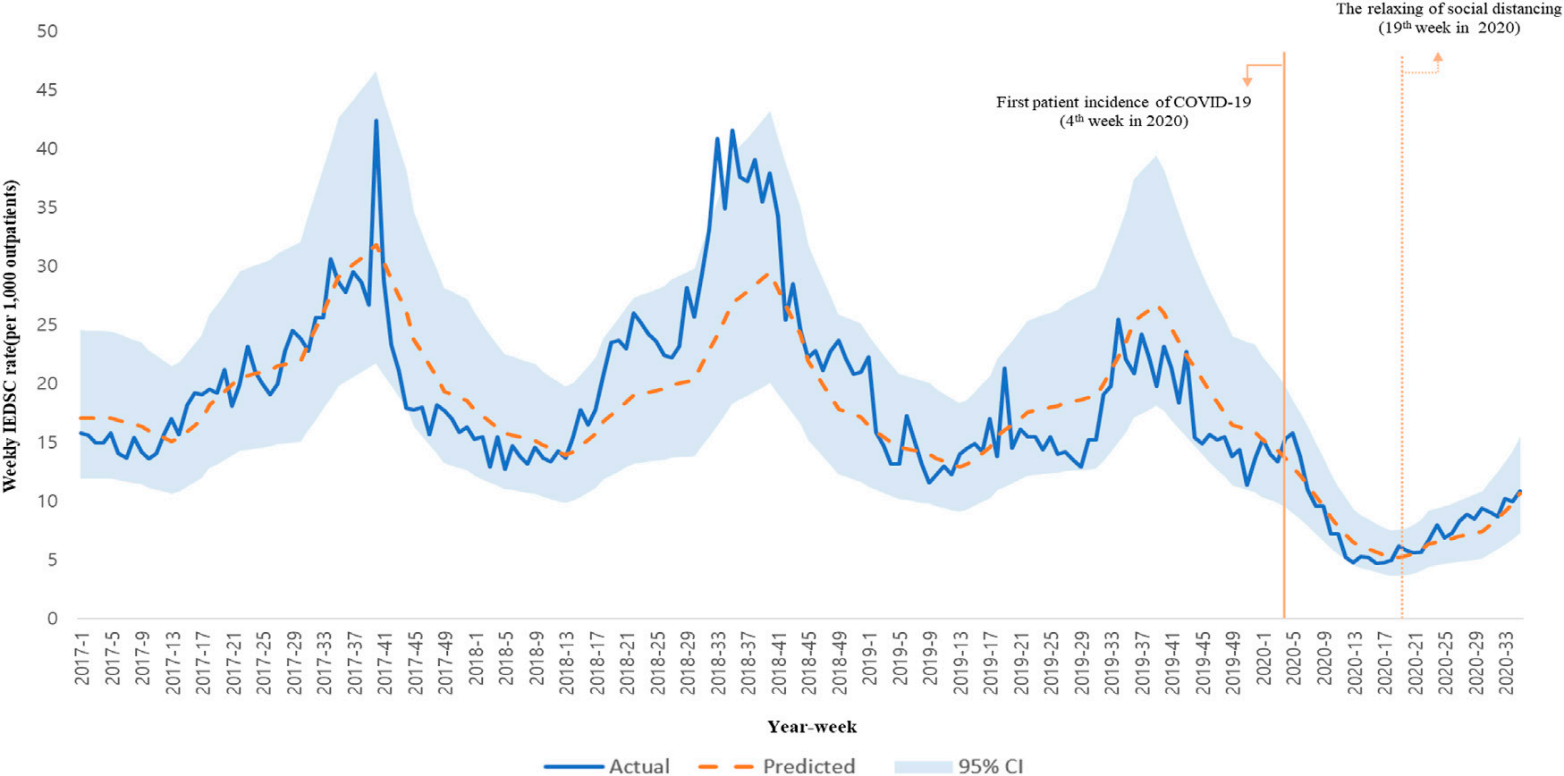
Total incidence of infectious eye disease suspected case actual data and adjusted mean data’s time trends (South Korea, 2017–2020).

Figure 2 shows a comparison between the prediction and actual values of our analysis model. The predicted values were accurately estimated (overall R^2^ = 0.86).

**FIGURE 3:**
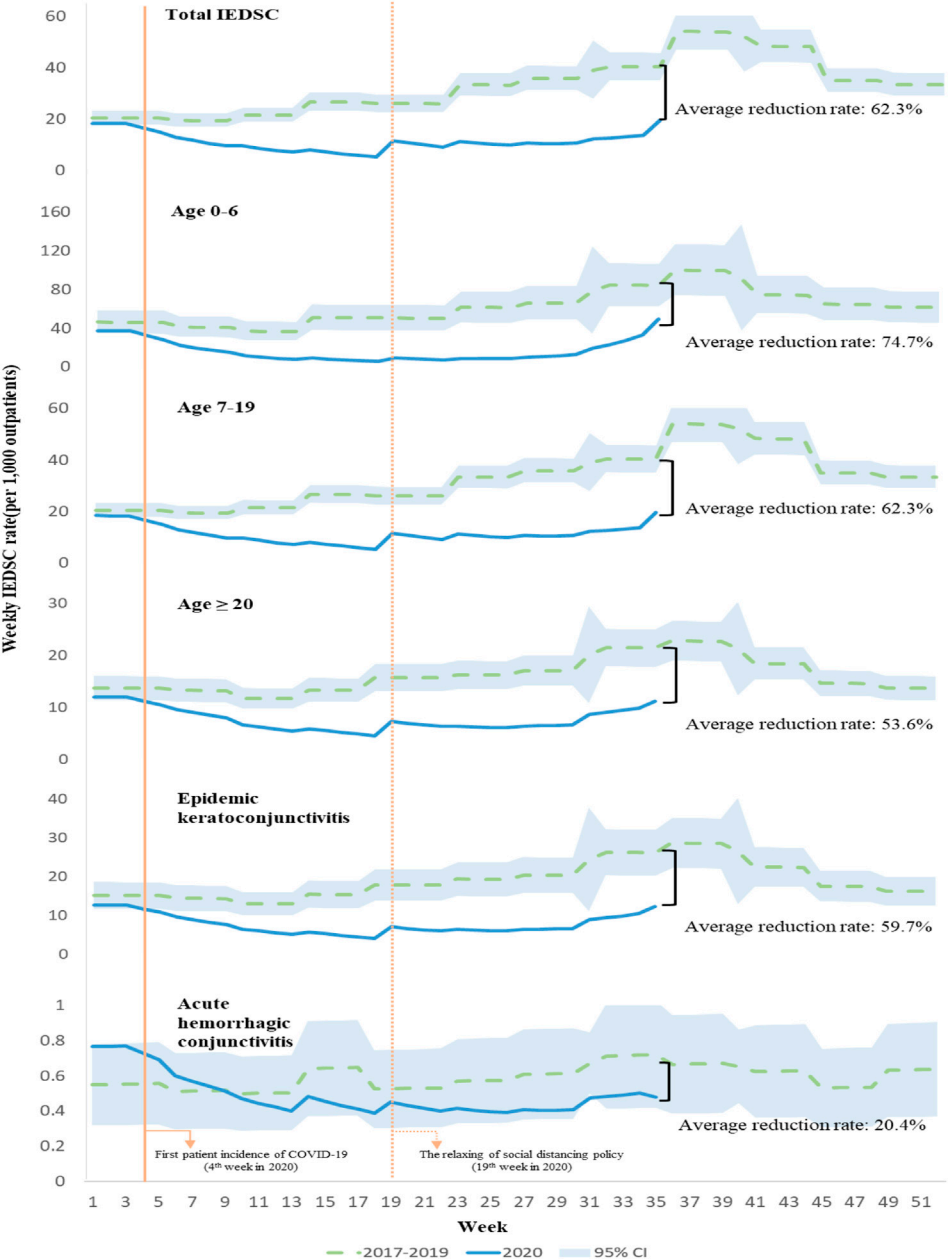
Weekly infectious eye disease suspected case incidence rate for all causes by age group and infection groups (South Korea, 2017–2020).

Regarding the changes in IEDSC rate in 2020 compared to changes in 2017–2019, the estimated average reduction in total IEDSC rate was 62.3% (Figure 3). The IEDSC rate in the age group 0–6 was reduced by 74.7% on average. The average reduction was 62.3%, 53.6%, and 59.7% in the age 7–19, age ≥20, and epidemic keratoconjunctivitis groups, respectively. Acute haemorrhagic conjunctivitis did not decrease significantly, with an average reduction rate of 20.4%.

Table 2 presents the marginal effects of the dependent variables. The first COVID-19 patient incidence decreased the IEDSC, and later increased due to the effect caused by the relaxation of the social distancing policy. Considering the effects of these two events, the IEDSC in all groups decreased.

**TABLE 2:** Marginal effects from results of segmented regression analysis (South Korea, 2017–2020).

IEDSC	The effect of the first COVID-19 patient incidence on August 4th week 2020 (compared to January 3rd week)	The effect of the relaxing of the social distancing policy on August 4th week 2020 (compared to April 5th week)	The effects of both policies on August 4th week 2020 (compared to January 3rd week 2020)
Total IEDSC	−2.288	0.493	−1.795
0–6 age	−4.304	0.849	−3.455
7–19 age	−3.168	0.966	−2.202
≥20 age	−2.003	0.420	−1.583
Epidemic keratoconjunctivitis	−2.355	0.514	−1.841
Acute haemorrhagic conjunctivitis	−1.789	0.454	−1.335

IEDSC, infectious eye disease suspected case.

## Discussion

### Key Findings

The current study used the KCDC’s nationwide infectious disease incidence monitoring data to investigate the impact of the social distancing policy implemented in the COVID-19 fundamental response strategy on IEDSC incidence in all age groups. Although a seasonal epidemic showed an increase in all IEDSCs from spring to summer in 2018–2019, the number of IEDSCs rapidly decreased in spring 2020 after implementing the social distancing policy and slightly increased in summer after the relaxing of the social distancing policy. This finding showed that social distancing effectively reduced the number of IED patients in all age groups.

### Interpretation

Most infections of viral conjunctivitis are transmitted by direct contact *via* contaminated fingers, swimming pool water, or infected patients in hospitals, kindergartens, and schools. Good hand hygiene and isolation from the infected patients are recommended to prevent the spread of acute viral conjunctivitis [17, 18]. The implementation of social distancing policy may prevent people from contacting IED patients or visiting public places such as swimming pools and hospitals; other public health interventions such as hand hygiene combined with social distancing policy may create a synergistic effect to decrease the number of IED patients [4, 17, 18]. Furthermore, postponing the start of kindergarten and school closures from February–March 2020 may also be helpful in preventing transmission of infectious conjunctivitis, especially childhood conjunctivitis [18, 26]. A previous study confirmed that social distancing at schools and daycare centre closures would substantially eliminate contact between children, significantly reducing multiple communicable diseases [10]. The rapid increase in the number of IED patients between 0 and 6 years after ending the social distancing policy, as observed in this study, also supports the relationship between social distancing and childhood conjunctivitis. Therefore, the study findings strengthen the evidence linking the implementation of social distancing policies to a decrease in the risk of acute conjunctivitis.

However, there are concerns regarding the interpretation of the findings in this study. Previous research has reported that an increase in particulate matter exposure, such as PM_10_ and PM_2.5_ was associated with an increased risk of acute conjunctivitis in Singapore and China [27, 28]. According to a report on air quality in South Korea, air pollution, especially with PM_10_ and PM_2.5_, in February–May 2020 was lower than the air pollution of the same month in 2017–2019; while the air pollution in June 2020 was similar to that of the same month in 2017–2019 [29]. This was observed because social distancing policies may have suppressed social activities and reduced traffic, which led to decreased air pollutant concentrations, especially in urban areas. China has also reduced industrial activities and travel, closed schools, and established numerous quarantine stations to reduce the spread of the illness after the outbreak of COVID-19 [30]. As a result, it was confirmed that the concentration of NO_2_ (nitrogen dioxide) in fossil fuels in February 2020 was lower than that in February 2019, and air pollution was reduced [31]. The trend of air pollution may also influence the incidence of IED outpatients; therefore, further research should include air pollution as a risk factor. As another concern, a systematic review reported that conjunctivitis can be a symptom of COVID-19 [32]. A thorough differential diagnosis of the virus is necessary for infected conjunctivitis because the possibility of viral infection due to COVID-19 should not be ruled out.

In general, most cases of keratoconjunctivitis and acute haemorrhagic conjunctivitis occur in summer, around July–September [33, 34]. Interestingly, in this study, social distancing showed substantial and consistent efficacy in suppressing the transmission of keratoconjunctivitis compared with that of acute haemorrhagic conjunctivitis. Furthermore, the number of keratoconjunctivitis outpatients rapidly increased compared to that of acute haemorrhagic conjunctivitis outpatients after relaxing the social distancing policy. This difference in the number of keratoconjunctivitis and acute haemorrhagic conjunctivitis outpatients may be because keratoconjunctivitis is majorly caused by adenovirus, while acute haemorrhagic conjunctivitis is majorly caused by enterovirus [17–19]. Previous research has shown that keratoconjunctivitis may be affected by humidity, rainfall, and wind speed rather than by temperature [35]. In 2020, the weather in South Korea showed the longest rainy season with high humidity and torrential downpours from the end of June to mid-August [36]. Therefore, environmental factors should be considered in further research to investigate the patterns of the number of keratoconjunctivitis and acute haemorrhagic conjunctivitis outpatients.

### Strengths and Limitations

This study investigated the impact of social distancing policies on the number of IED patients. The study findings provide evidence that appropriate social distancing can prevent the transmission of IED, especially in children and adolescents. This study had some limitations. First, this study included only viral conjunctivitis and did not include other common types of conjunctivitis, such as those of bacterial and allergenic origin. Therefore, the results cannot be generalized to individuals with other types of conjunctivitis. Second, this study did not adjust for the effects of air quality and region (urban versus country) as a risk factor for the number of IED patients, which limits the interpretation of the findings.

### Conclusion

In this study, the COVID-19 public health response and social distancing policy were found to have an indirect impact on the development of IED among children and adolescents. As a result, post-COVID-19 period also needs maintenance of personal hygiene measures, such as hand hygiene after coughing. In addition, we hope that national-level interventions such as reducing air pollution, along with the precautionary measures will significantly reduce the financial burden of developing infectious ophthalmic diseases.
